# The upper limit of cardiorespiratory fitness associated with longevity: an update

**DOI:** 10.3934/publichealth.2019.3.225

**Published:** 2019-07-04

**Authors:** Johannes Burtscher, Gerhard Ruedl, Markus Posch, Klaus Greier, Martin Burtscher

**Affiliations:** 1 Laboratory of molecular and chemical biology of neurodegeneration, École Polytechnique Fédérale de Lausanne (EPFL), Switzerland; 2 Department of Sport Science, University of Innsbruck, Austria; 3 Department of Physical Education, Private University of Education (KPH-ES) Stams, Austria

**Keywords:** fitness, cardiorespiratory, VO_2_max, longevity, mortality

## Abstract

In 2013, mortality reductions with improving cardiorespiratory fitness (CRF) have been suggested to persist until 13 METs. More recently, accumulating evidence from large-scale studies suggests that mortality from all causes decreases with increasing CRF levels, apparently without upper limit of CRF. However, when baseline CRF is assessed in later life, upper limits of CRF decrease depending on the individual fitness level at baseline and the volume and intensity of physical activity performed during follow up. Consequently, both a CRF level as high as possible during early adulthood, achieved by appropriate exercise interventions, and a small CRF decline during later life, by continuation of regular physical activity, will help to optimize longevity.

## 1. Introduction

High cardiorespiratory fitness (CRF), expressed as maximal aerobic capacity (VO_2_max), is accompanied by a reduced risk of death, independent of age, sex, ethnicity and comorbidities [1]. CRF is considered as one of the most important mortality predictors, highlighting the great importance of exercise testing in clinical practice [2]. Ten years ago, results of a meta-analysis reported that increasing the aerobic capacity by 1 metabolic equivalent (1 MET = 3.5 mL resting oxygen consumption per minute and kg body mass) is associated with an about 13% decrease of mortality risk [3]. In this study high CRF was set to ≥ 10.9 METs without further definition of an upper limit of benefit regarding longevity. In 2013, mortality reductions with improving CRF have been suggested to persist until 13 METs [4]. More recently, accumulating evidence from large-scale studies suggests that mortality from all causes decreases with increasing CRF levels, apparently without upper limit of CRF [1],[5]–[7].

## 2. Relevance of age when assessing CRF levels

“Without upper limit” deserves further consideration. CRF peaks between the 2nd and 4th decade and then inevitably declines in sedentary and trained individuals as well [8]. Thus, the time of CRF assessment during lifespan is crucial for the evaluation of an upper CRF limit associated with maximal benefits on longevity. Such a limit can be assumed to be higher when assessed at an age of individual performance peak and may differ between sexes. Recent studies assessed CRF in large cohorts [1],[5]–[7] somewhen during midlife (42.8 ± 12.2 years up to 53.4 ± 12.6 years) with various follow-up periods (8.4 to 46 years) (Table 1). All these studies demonstrated decreasing all-cause mortality with increasing CRF, in one study extending to individuals up to an exercise capacity of about 16 METs [5]. The authors of that study however, admitted lack of sufficient power to model subjects with a CRF above 16 METs. In the long-term follow-up study by Clausen et al. [6], a low versus high CRF was associated with a longevity benefit of about 5 years. In the other studies, the reduction in mortality risk due to a CRF increase by 1 MET varied between 10% and 15% [1],[5],[7]. Variation may be explained by differences regarding the time when baseline values are taken, the sex distribution, ethnicity, etc.. Therefore, the assessment of an existing upper CRF limit for longevity requires the inclusion of subjects at an age when individual CRF peaks.

## 3. Association between the decline of CRF with aging and all-cause mortality

Importantly, the “elite level” of CRF, pre-defined by Mandsager and colleagues, decreased in males from ≥ 16.3 to ≥ 10 METs and in females from ≥ 15.0 to ≥ 8.4 METs with aging (18 to over 80 years) being for instance, ≥ 14 METs for males and ≥ 13 METs for females aged between 50 and 59 years [1]. It should be pointed out that much higher age-dependent levels can be achieved by real elite endurance athletes of both sexes [8]. Hence, it remains to be elucidated whether the gap between the “elite” aerobic capacity defined by Mandsager and colleagues [1] and the higher capacity attained by real elite endurance athletes [8] yields additional benefits with regard to healthy aging and longevity. When CRF is assessed in later life, upper limits of CRF decrease depending on the individual fitness level at baseline and the volume and intensity of physical activity performed during follow up [8]. However, only a very few studies assessed long-term changes in CRF and the related mortality risk during follow up. For instance, Laukkanen and colleagues determined VO_2_max in a population-based sample of 579 men (42 to 60 years) at baseline and repeated exercise testing 11 years later [9]. CRF decreased by an average of 1.5 METs and a lesser decrease of 1 MET was associated with a 31.5% risk reduction of all-cause mortality, impressively demonstrating the importance of maintaining fitness.

**Table 1.** Recent studies evaluating the association between cardiorespiratory fitness (CRF) and mortality/longevity.

Publication	Study population (n)	Age mean, SD (years)	Follow up (years)	Reference CRF (METs)	High CRF (METs)	Mortality decrease or longevity benefit (High CRF vs. Reference CRF)
	Males (%)					
	Type of population					
Feldman et al. 2015	37,855	49.6 ± 11	11.5	10–11	≥ 14 *	−79%
	63.7					
	adults free from CVD					
Clausen et al. 2018	5,107	48.8 ± 5.4	46	5.9	14.2	+4.9 years
	100					longevity benefit
	adult males free from CVD					
Imboden et al. 2018	4,137	42.8 ± 12.2	24.2	8.3 (males)	14.2	−73%
	56			6.1 (females)	10.3	−43%
	apparently healthy adults					
Mandsager et al. 2018	122,007	53.4 ± 12.6	8.4	8.2	13.8	−75%
	59.2					
	adults without and with comorbidities					

Note: MET: metabolic equivalent; 1 MET = 3.5 mL resting oxygen uptake per minute and kg body mass;

CVD: cardiovascular disease: * probability of death was decreasing up to 16 METs.

## 4. Conclusion

Taken together, both a CRF level as high as possible during early adulthood, achieved by appropriate exercise interventions, and a small CRF decline during later life, by continuation of regular physical activity, will help to optimize longevity.
